# Supplementary material for: Climate warming and elevated CO2 alter peatland soil carbon sources and stability
Source: Nat Commun. 2023 Nov 20;14:7533. doi: 10.1038/s41467-023-43410-z (PMC10662476; doi:10.1038/s41467-023-43410-z)
Supplement: Supplementary file 1 — Supplementary Information [file 41467_2023_43410_MOESM1_ESM.pdf]

Climate warming and elevated CO_2 alter peatland soil carbon sources and stability

Nicholas O.E. Ofiti[1,2], Michael W.I. Schmidt[1], Samuel Abiven[2,3], Paul J. Hanson[4], Colleen M. Iversen[4], Rachel M. Wilson[5], Joel E. Kostka[6], Guido L.B. Wiesenberg[1], Avni Malhotra[1,7]

[1]Department of Geography, University of Zurich, Zurich, Switzerland. [2]CEREEP-Ecotron Ile De France, ENS, CNRS, PSL Research University, Saint-Pierre-lès-Nemours, France. [3]Laboratoire de Géologie, Département de Géosciences, Ecole normale supérieure (ENS), Paris, France. [4]Environmental Sciences Division and Climate Change Science Institute, Oak Ridge National Laboratory, Oak Ridge, TN, USA. [5]Department of Earth, Ocean and Atmospheric Sciences, Florida State University, Tallahassee, Florida, USA. [6]School of Biological Sciences and School of Earth and Atmospheric Sciences, Center for Microbial Dynamics and Infection, Georgia Institute of Technology, Atlanta, Georgia, USA,[7]Current address: Biological Sciences Division, Pacific Northwest National Laboratory, Richland, Washington, USA.

Supplemental Materials:

Supplementary Table S1. Environmental conditions in the experimental enclosures[1].

Plot#	CO_2 treatment	Nominal warming	CO_2 concentration (ppm)	Soil temperature (°C)	ANPP	BNPP	NPP	Fine root biomass (g/m²)	Depth to water table (cm)
6	Ambient	+0	423	4.0	317.3	67.7	385.0	54.7	16.9
19	Elevated	+0	708	5.5	259.5	25.7	285.2	23.1	27.9
20	Ambient	+2.25	415	6.9	320.9	90.6	411.5	171.8	18.1
11	Elevated	+2.25	698	7.1	258.9	38.6	297.5	58.3	26.7
13	Ambient	+4.5	415	8.1	229.5	87.1	316.6	91.3	32.1
4	Elevated	+4.5	723	8.8	196.5	106.9	303.4	281.5	23.1
8	Ambient	+6.75	416	9.6	246.5	90.3	336.8	264.2	37.4
16	Elevated	+6.75	743	10.4	170.9	95.0	265.9	260.4	24.9
17	Ambient	+9	429	11.4	126.0	99.0	225.0	326.7	43.9
10	Elevated	+9	736	12.1	234.8	116.25	351.1	321.2	45.0

Note: Soil temperature was measured at -0.3 m below the hollows averaged over the period 2016 to 2018. Aboveground net primary productivity (ANPP), belowground net primary productivity (BNPP), and net primary productivity (NPP) data is from ref[2], fine-root biomass data is from ref[3], and maximum depth to water table data is from ref[1].

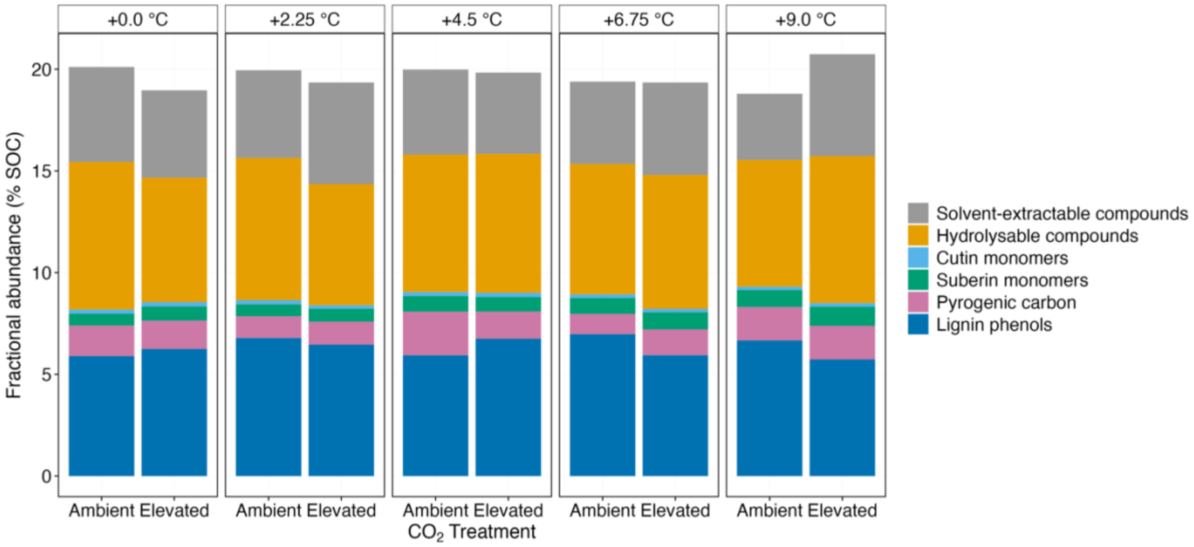

Supplementary Figure 1. The relative abundance of SOC components as a fraction of SOC. Relative proportion of total solvent-extractable compounds (free lipids), ester-bound hydrolysable compounds (non-specific monomers), cutin and suberin (specific) monomers, pyrogenic carbon, and lignin phenols normalized to organic carbon concentration in the surface peat (0-30 cm depth; Mean, *n* = 3) following 4 years of warming and 2 years of elevated atmospheric CO_2 concentrations.

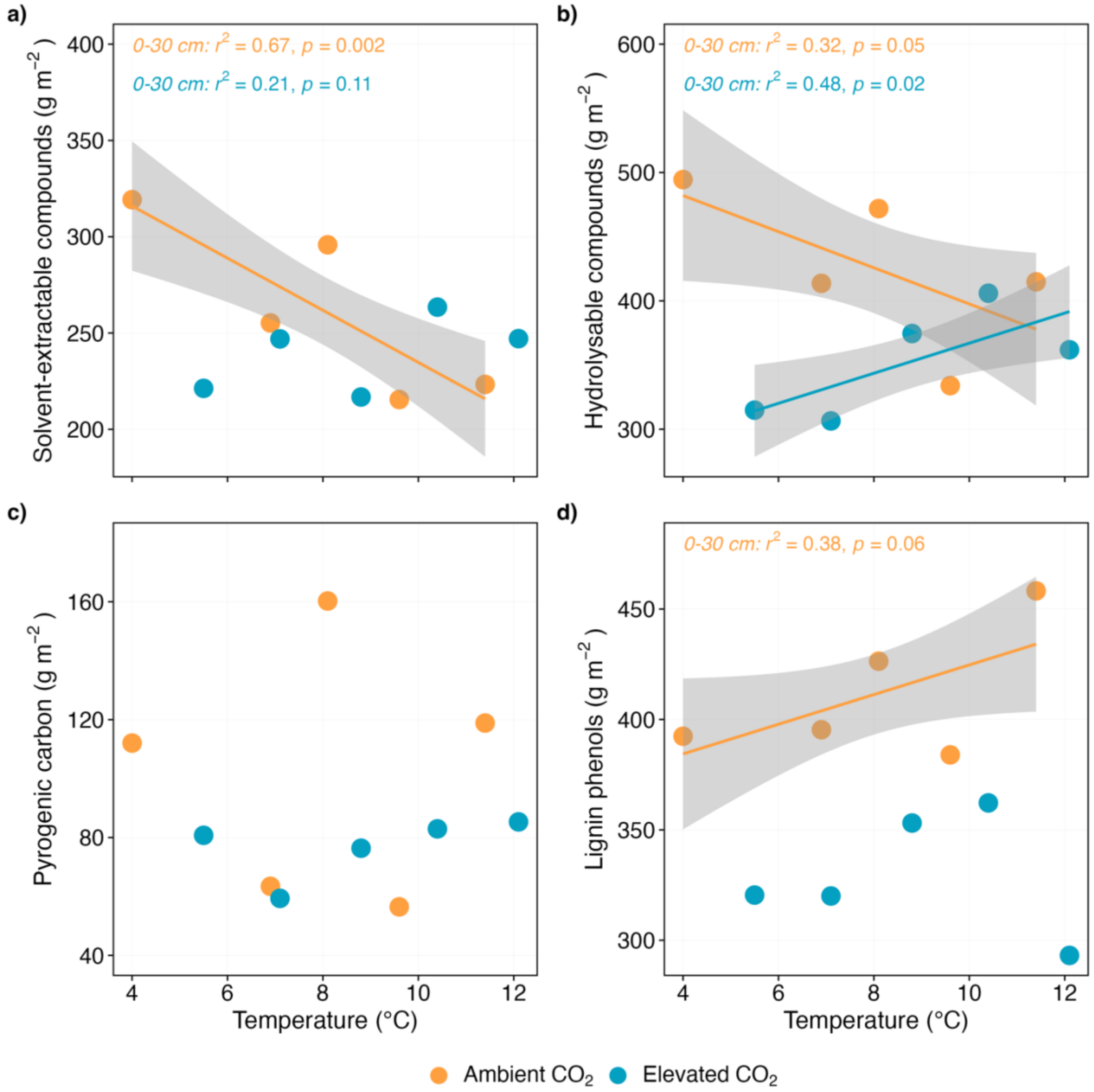

Supplementary Figure 2. The absolute concentration of plant-, microorganism- and fire-derived SOC components. Linear temperature response of the total sum of a) solvent-extractable compounds (free lipids), b) hydrolysable compounds (ester-bound lipids), c) pyrogenic carbon and d) lignin phenols in the surface peat following 4 years of warming and 2 years of elevated atmospheric CO_2 concentrations. The stocks are plotted against average soil temperature measured at 0.3 m below the hollow surface from 2016 to 2018. Colours represent ambient (orange) or elevated CO_2 (blue) treatment (n = 5 per treatment). Lines indicate significant treatment effects $p < 0.05$. Linear regression with 95% confidence intervals is shown in grey. The absence of a line and/or confidence intervals indicates no significant trend. Bulk density (g soil cm^{-3}) was estimated as mean bulk density of pre-treatment (2012) and 2020 peat sampling (see Supplementary Figure 7). We calculated stocks by multiplying bulk density value by total concentration of individual SOC component (mg g $peat^{-1}$) and summing up the stocks over the entire depth interval (0-30 cm depth; n = 3).

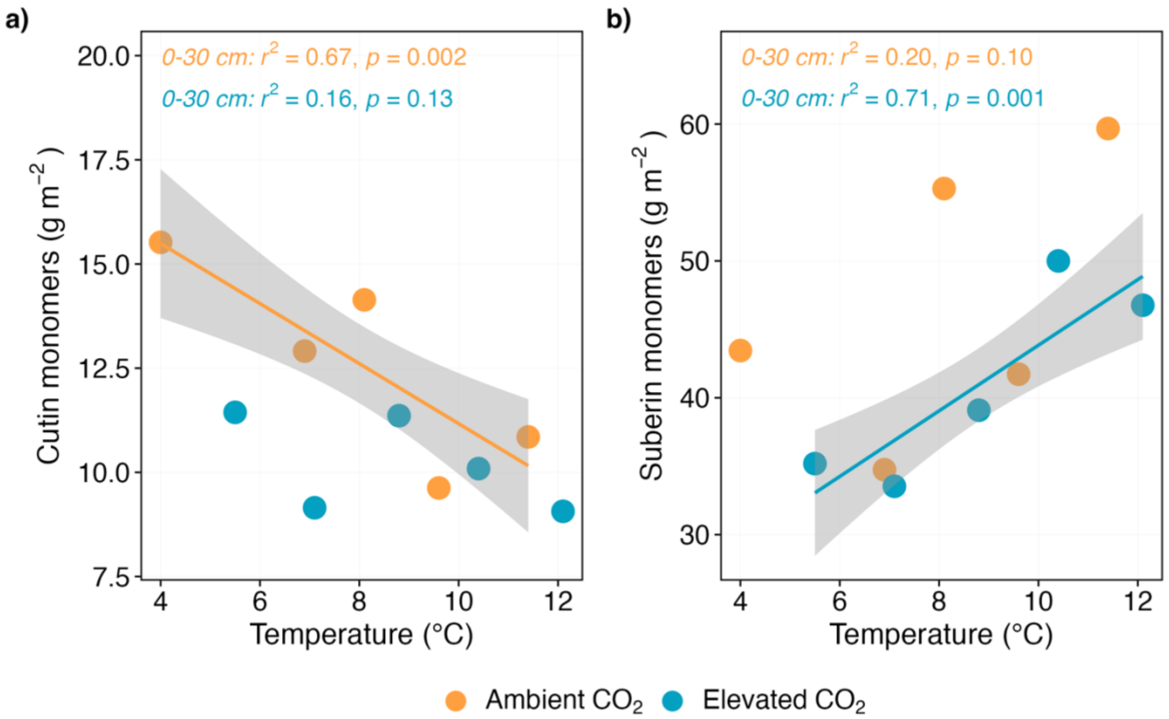

Supplementary Figure 3. The absolute concentration of SOC components distinct to leaf/needle and root compartment. Linear temperature response of the total sum of monomers distinct to a) leaf/needle (cutin), and b) root/bark (suberin) compartment in the surface peat following 4 years of warming and 2 years of elevated atmospheric CO_2 concentrations. The stocks are plotted against average soil temperature measured at 0.3 m below the hollow surface from 2016 to 2018. Colours represent ambient (orange) or elevated CO_2 (blue) treatment (n = 5 per treatment). Lines indicate significant treatment effects $p < 0.05$. Linear regression with 95% confidence intervals is shown in grey. The absence of a line and/or confidence intervals indicates no significant trend. Bulk density (g soil cm^{-3}) was estimated as mean bulk density of pre-treatment (2012) and 2020 peat sampling (see Supplementary Figure 7). We calculated stocks by multiplying bulk density value by total concentration of cutin and suberin (mg g $peat^{-1}$) and summing up the stocks over the entire depth interval (0-30 cm depth; n = 3).

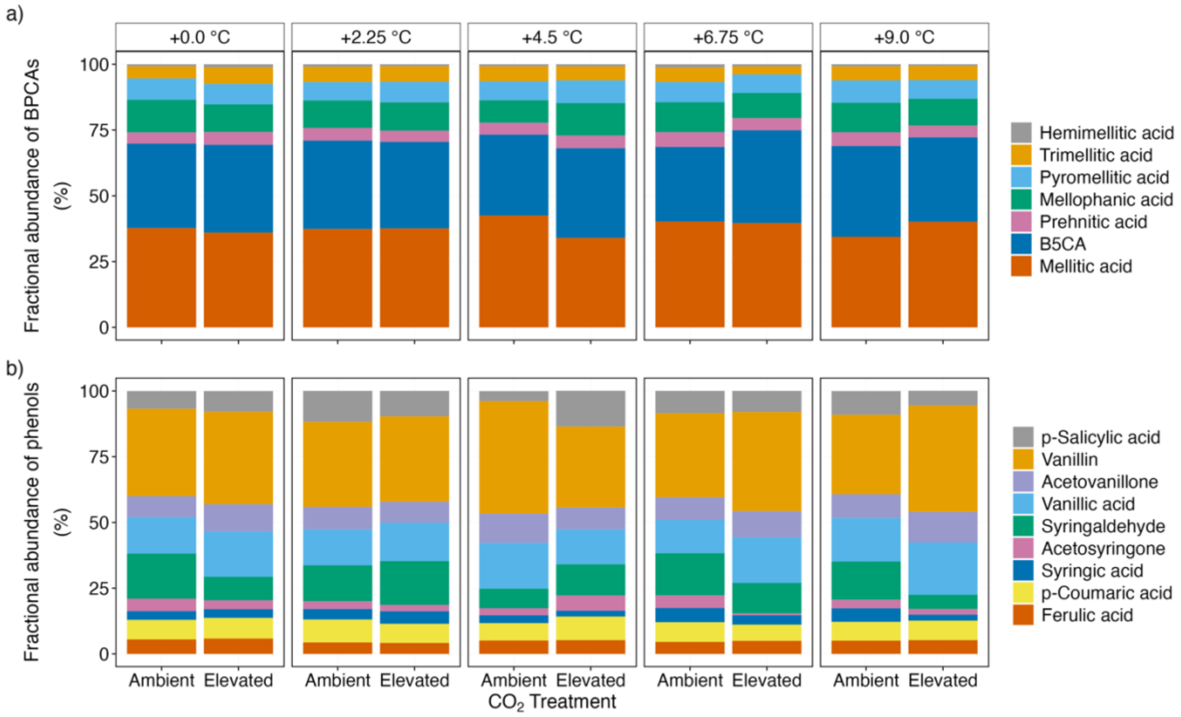

Supplementary Figure 4. The relative abundance of polymeric SOC. Relative proportion of a) pyrogenic carbon (PyC) polycyclic structures and b) lignin phenols target compounds normalized to total concentrations of PyC and lignin respectively, in the surface peat (0-30 cm depth; Mean, *n* = 3) following 4 years of warming and 2 years of elevated atmospheric CO_2 concentrations.

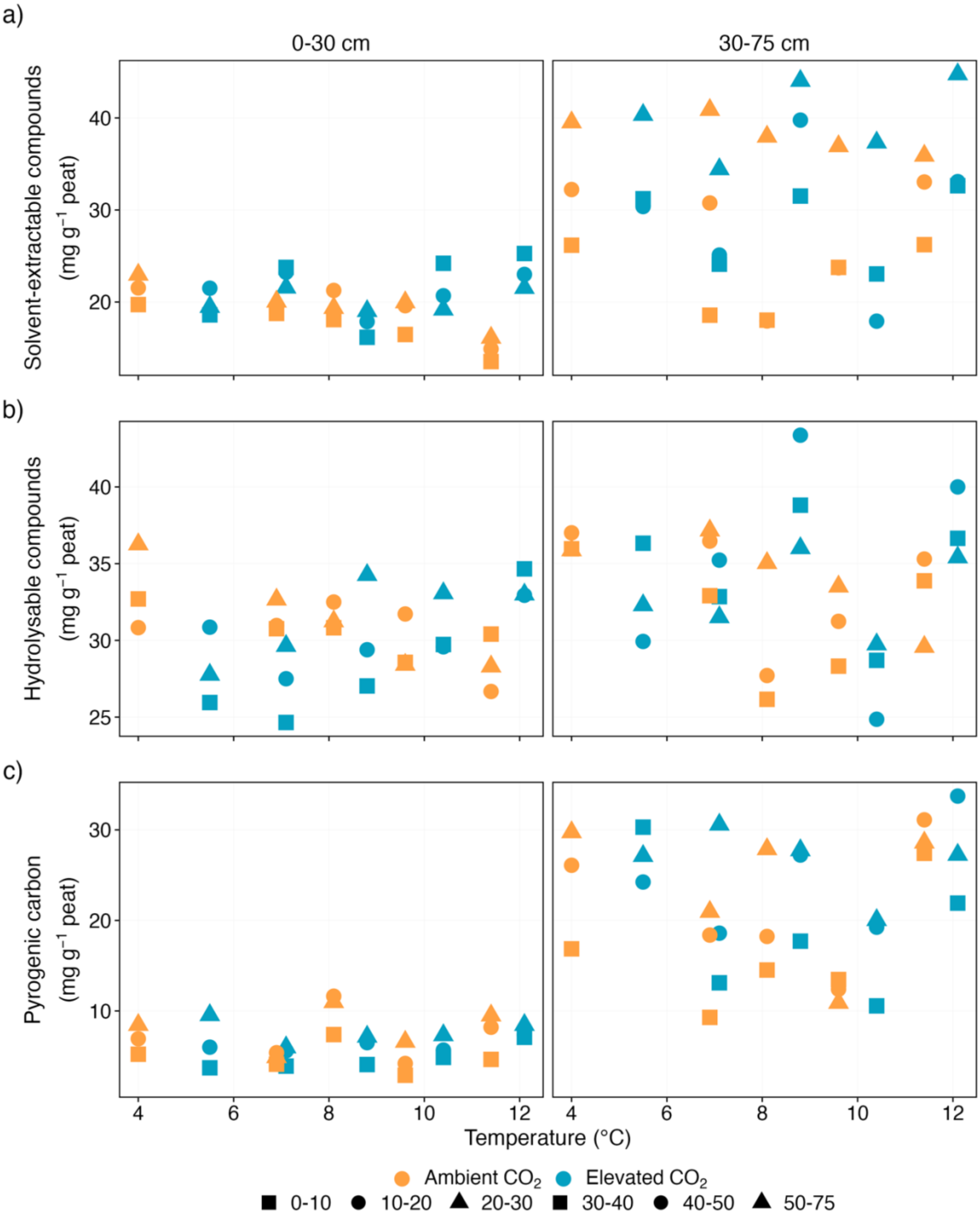

Supplementary Figure 5. The concentration of plant-, microorganism-, and fire-derived SOC components. Linear temperature response of the total sum of a) solvent-extractable compounds (free lipids), b) hydrolysable compounds (ester-bound lipids), and c) pyrogenic carbon in the surface peat (0-30 cm depth) and deeper peat (30-75 cm depth) following 4 years of warming and 2 years of elevated atmospheric CO_2 concentrations. The concentrations are plotted against average soil temperature measured at 0.3 m below the hollow surface from 2016 to 2018. Colours represent ambient (orange) or elevated CO_2 (blue) treatment (*n* = 5 per treatment). Symbols represent different sampling depths.

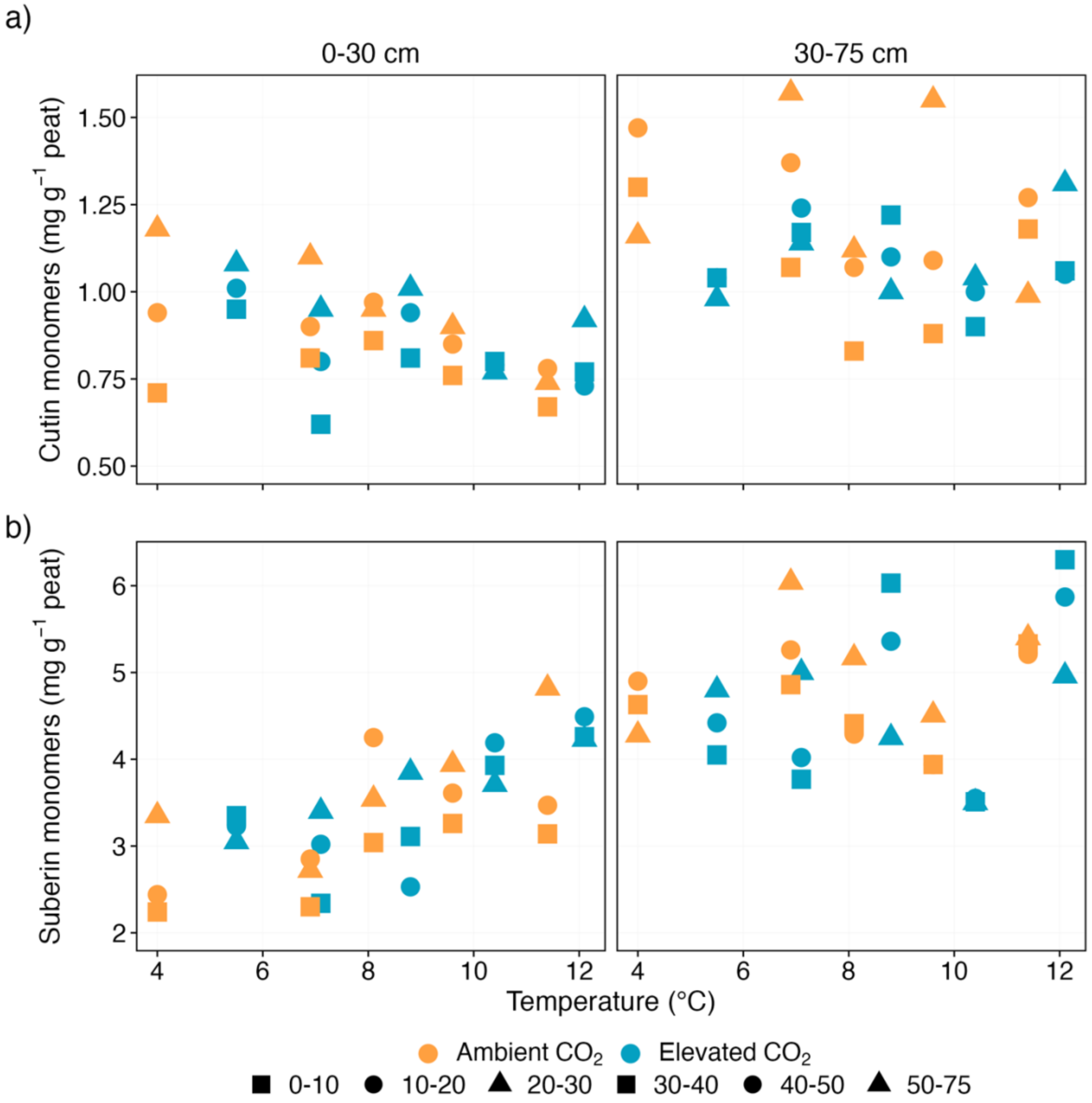

Supplementary Figure 6. The concentration of SOC components distinct to leaf/needle and root compartment. Linear temperature response of the total sum of monomers distinct to a) leaf/needle (cutin), and b) root/bark (suberin) compartment in the surface peat (0-30 cm depth) and deeper peat (30-75 cm depth) following 4 years of warming and 2 years of elevated atmospheric CO_2 concentrations. The concentrations are plotted against average soil temperature measured at 0.3 m below the hollow surface from 2016 to 2018. Colours represent ambient (orange) or elevated CO_2 (blue) treatment (n = 5 per treatment). Symbols represent different sampling depths.

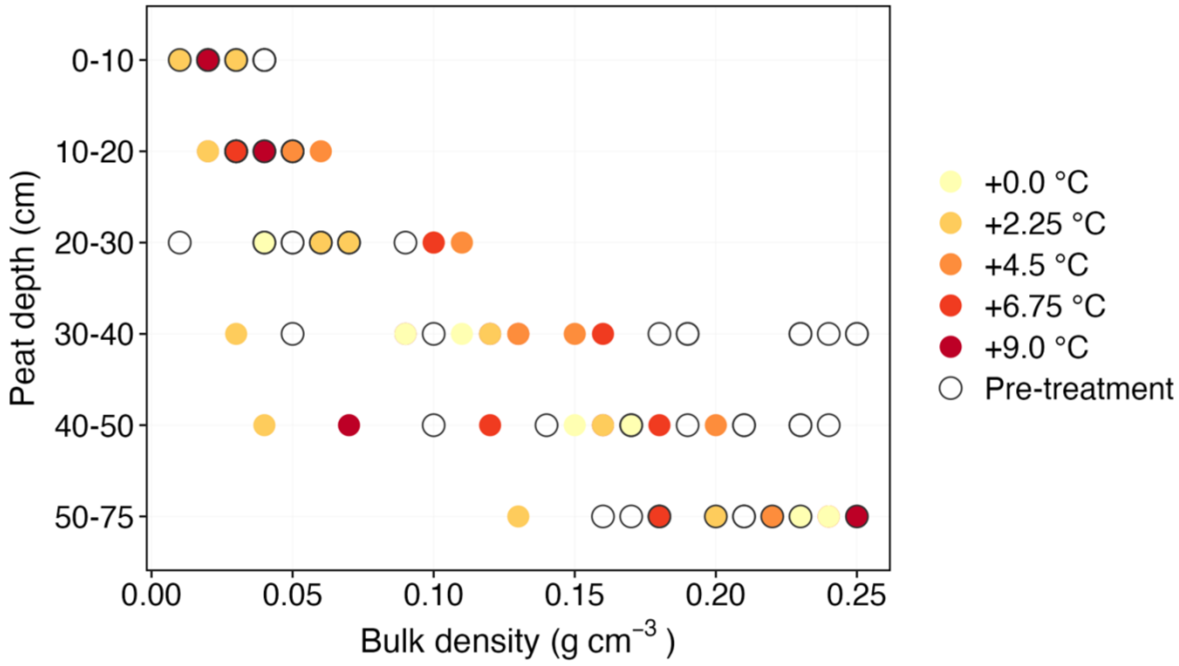

Supplementary Figure 7. Bulk density measurements[1]. Bulk density values (g peat cm^{-3}) from pre-treatment (2012; open black symbols) and 2020 peat sampling (closed colored symbols) to a depth of 75 cm. Overall, pre-treatment bulk density measurements did not differ significantly from 2020 measurements in ambient CO_2 or elevated CO_2 plots.

Supplementary Table S2. Hydrolysable compounds (ester-bound lipids) released from *Picea mariana*, *Larix laricina*, *Rhododendron groenlandicum*, *Chamaedaphne calyculata*, and *Sphagnum* mosses (μg g^{-1} dry weight) after alkaline hydrolysis of leaf/needle, stem/bark and root material from the SPRUCE control plot (n.d., below detection limit). Mean values were calculated for the individuals assessed, n= 6 for shrubs, n = 2 for trees and n = 20 for *Sphagnum* mosses shoots. Specific monomers (in bold) for cutin are hexacosanoic acid (*n*-$C_{26:0}$), octacosanoic acid (*n*-$C_{28:0}$), Dihydroxyoctadecanoic acid (9,ω-dihydroxy $C_{18:0}$) and Dihydroxyoctadecenoic acid (x,ω-dihydroxy $C_{18:1}$) and specific suberin monomers are ω-hydroxy alkanoic acids with a chain length of C_{20}, C_{24} and C_{26} and α,ω-alkanedioic acid with a chain length C_{20}, C_{22} and C_{24} (also α,ω-Octadecenoic acids, $C_{18:1}$ diacid).

Compounds	*Larix laricina*			*Picea mariana*			*Rhododendron groenlandicum*			*Chamaedaphne calyculata*			*Sphagnum* mosses
	Needle	Stem	Root	Needle	Stem	Root	Leaf	Stem	Root	Leaf	Stem	Root	
n-Alcohols													
n-$C_{16:0}$	46	27	127	121	31	210	74	11	121	43	11	108	5
n-$C_{18:0}$	37	23	64	29	98	267	33	15	73	28	16	74	2
n-$C_{20:0}$	55	55	470	74	184	699	32	55	124	30	51	144	n.d.
n-$C_{22:0}$	165	207	543	200	367	822	141	109	488	167	110	466	25
n-$C_{24:0}$	263	63	732	330	118	1150	329	44	611	236	57	723	19
n-$C_{26:0}$	26	38	182	33	57	247	27	28	191	29	33	183	2
n-Alkanoic acids													
n-$C_{14:0}$	157	25	51	182	19	50	231	21	72	326	20	58	6
n-$C_{15:0}$	6	6	9	7	4	11	14	6	9	9	9	6	n.d.
n-$C_{16:1}$	42	4	78	40	4	91	46	4	84	60	5	84	2
n-$C_{16:0}$	2742	309	863	2355	387	688	5508	441	968	4630	431	733	380
n-$C_{17:0}$	n.d.	n.d.	5	5	n.d.	n.d.	n.d.	4	n.d.	5	6	n.d.	n.d.
n-$C_{18:2}$	168	32	46	223	32	47	296	23	31	397	16	34	2
n-$C_{18:1}$	466	127	206	358	85	151	625	81	177	684	109	187	42
n-$C_{18:0}$	1951	137	377	1940	172	362	2258	155	286	2617	183	298	169
n-$C_{20:0}$	397	29	36	391	25	38	394	28	41	545	27	30	4
n-$C_{21:0}$	7	n.d.	5	4	n.d.	4	n.d.	n.d.	n.d.	5	n.d.	n.d.	n.d.
n-$C_{22:0}$	175	33	53	181	32	52	230	26	50	268	26	50	16
n-$C_{23:0}$	6	n.d.	3	5	n.d.	3	5	n.d.	4	6	n.d.	n.d.	n.d.
n-$C_{24:0}$	1347	50	302	1298	65	263	1923	56	224	2595	91	236	8
n-$C_{25:0}$	n.d.	n.d.	n.d.	n.d.	n.d.	n.d.	4	n.d.	n.d.	5	n.d.	n.d.	n.d.
***n*-$C_{26:0}$**	402	n.d.	n.d.	362	n.d.	n.d.	466	n.d.	n.d.	562	n.d.	n.d.	4
***n*-$C_{28:0}$**	199	n.d.	n.d.	171	n.d.	n.d.	222	n.d.	n.d.	253	n.d.	n.d.	n.d.
α-Hydroxy alkanoic acids													
α-$C_{24:0}$	150	72	322	293	128	264	211	96	198	279	59	213	n.d.

α-$C_{26:0}$	48	16	58	70	12	40	67	10	41	88	27	48	8
ω-Hydroxy alkanoic acids													
ω-$C_{16:0}$	3658	864	8762	3245	749	10981	3813	921	12928	3211	999	10277	10
ω-$C_{18:1}$	2856	407	4964	2720	473	5742	2852	591	1582	1964	625	3368	6
ω-$C_{18:0}$	705	362	2508	710	387	2691	1273	398	1633	717	347	1174	6
**ω-$C_{20:0}$**	n.d.	n.d.	3730	n.d.	n.d.	7033	n.d.	n.d.	2199	n.d.	n.d.	2258	n.d.
ω-$C_{22:0}$	155	72	2520	166	126	2873	174	113	2245	210	155	2966	7
**ω-$C_{24:0}$**	n.d.	n.d.	377	n.d.	n.d.	502	n.d.	n.d.	301	n.d.	n.d.	333	n.d.
**ω-$C_{26:0}$**	n.d.	n.d.	269	n.d.	n.d.	223	n.d.	n.d.	184	n.d.	n.d.	203	n.d.
α,ω-Alkanedioic acids													
α,ω-$C_{10:0}$	24	6	29	28	n.d.	32	24	5	21	24	3	24	n.d.
α,ω-$C_{11:0}$	n.d.	5	11	4	6	11	n.d.	8	9	n.d.	5	12	4
α,ω-$C_{14:0}$	244	54	244	270	66	265	254	51	250	203	48	239	40
α,ω-$C_{16:0}$	506	56	1373	493	67	806	243	68	943	436	40	1026	7
α,ω-$C_{18:0}$	265	26	2535	484	16	2183	443	12	1036	252	9	1292	4
**α,ω-$C_{18:1}$**	n.d.	n.d.	4113	n.d.	n.d.	3836	n.d.	n.d.	3799	n.d.	n.d.	4704	n.d.
**α,ω-$C_{20:0}$**	n.d.	n.d.	3445	n.d.	n.d.	3926	n.d.	n.d.	3193	n.d.	n.d.	4910	n.d.
**α,ω-$C_{22:0}$**	n.d.	n.d.	1113	n.d.	n.d.	979	n.d.	n.d.	1158	n.d.	n.d.	1311	n.d.
**α,ω-$C_{24:0}$**	n.d.	n.d.	412	n.d.	n.d.	385	n.d.	n.d.	474	n.d.	n.d.	554	n.d.
Mid-chain hydroxy and epoxy alkanoic acids													
x,ω-dihydroxy $C_{16:0}$	2321	22	8	2570	7	9	8876	18	35	7962	24	44	6
**x,ω-dihydroxy $C_{18:1}$**	1435	n.d.	n.d.	1201	n.d.	n.d.	2518	n.d.	n.d.	3168	n.d.	n.d.	7
**9,ω-dihydroxy $C_{18:0}$**	267	n.d.	n.d.	322	n.d.	n.d.	307	n.d.	n.d.	597	n.d.	n.d.	4
9,10-epoxyω-hydroxy $C_{18:0}$	866	71	73	668	86	61	1451	126	70	1643	97	63	5
9,10,ω-trihydroxy $C_{18:0}$	1305	41	131	1537	65	159	17944	66	184	9558	84	183	2

Supplementary Table S3. Slopes of linear regression model when predicting solvent-extractable compounds (free lipids), hydrolysable compounds (ester-bound lipids), monomers distinct to leaf/needle (cutin), and root/bark (suberin) compartment, pyrogenic carbon, and lignin phenols against soil temperature. Each reported slope is from a single bivariate linear regression of each compound regressed against temperature under ambient or elevated CO_2 treatment in the surface peat (0-30 cm depth) following 4 years of warming and 2 years of elevated atmospheric CO_2 concentrations.

**Response variable**	**CO_2 treatment**	**Intercept**	**Slope estimate**	**Std. Error**	***P* value**
Solvent-extractable compounds	Ambient	25.08	-0.79	0.17	0.0004
	Elevated	18.21	0.32	0.29	0.28
Hydrolysable compounds	Ambient	36.07	-0.65	0.17	0.002
	Elevated	22.41	0.86	0.26	0.006
Cutin monomers	Ambient	1.10	-0.03	0.01	0.046
	Elevated	1.08	-0.02	0.01	0.07
Suberin monomers	Ambient	1.86	0.18	0.06	0.01
	Elevated	1.78	0.20	0.05	0.002
Pyrogenic carbon	Ambient	6.52	0.03	0.28	0.922
	Elevated	4.47	0.20	0.18	0.304
Lignin phenols	Ambient	25.51	0.51	0.22	0.04
	Elevated	31.83	-0. 38	0.15	0.026

Supplementary Table S4. Optimal mixed-effects linear regression models to explain variation in solvent-extractable compounds (free lipids), hydrolysable compounds (ester-bound lipids), monomers distinct to leaf/needle (cutin), and root/bark (suberin) compartment, pyrogenic carbon, and lignin phenols. This table includes all possible predictors for SOC compound in either ambient or elevated CO_2 plots in the surface peat (0-30 cm depth) following 4 years of warming and 2 years of elevated atmospheric CO_2 concentrations. In the table, SOC denotes soil organic carbon concentration, soil N denotes nitrogen concentration, NPP denotes net primary productivity, and ANPP denotes aboveground net primary productivity. Models are reported for significance criteria with $\alpha < 0.05$. Values in parentheses are standard errors. Significant effects are marked with * for $p < 0.05$, ** for $P < 0.01$, *** for $p < 0.001$, and **** for $p < 0.0001$.

Response variable	CO_2 treatment	Significance criterion	Predictor	Coefficient	Predictor	Coefficient	Predictor	Coefficient	Predictor	Coefficient	Adjusted R^2
Solvent-extractable compounds	Ambient	$p < 0.01$	Intercept	16.72*** (2.64)	ANPP	0.02* (0.01)	Fine-root biomass	-0.01 (0.005)	--	--	0.67
	Elevated	$p < 0.01$	Intercept	-23.31 (15.80)	SOC	0.10* (0.03)	Water table depth	0.19* (0.08)	Soil N	-0.55 (0.37)	0.52
Hydrolysable compounds	Ambient	$p < 0.05$	Intercept	37.90*** (3.26)	Fine-root biomass	-0.02** (0.004)	Soil N	-0.32 (0.24)	--	--	0.51
	Elevated	$p < 0.05$	Intercept	-12.42 (16.89)	Fine-root biomass	0.01* (0.005)	SOC	0.07 (0.04)	Soil N	0.06 (0.35)	0.59
Cutin monomers	Ambient	$p < 0.05$	Intercept	1.01*** (0.06)	Fine-root biomass	-0.001* (0.000)	--	--	--	--	0.25
	Elevated	$p > 0.05$	Intercept	-5.27* (1.84)	Water table depth	-0.30* (0.10)	Fine-root biomass	0.21* (0.07)	ANPP	0.71* (0.24)	0.44
Suberin monomers	Ambient	$p < 0.05$	Intercept	1.81** (0.42)	Water table depth	0.06* (0.02)	Fine-root biomass	-0.002 (0.002)	--	--	0.45
	Elevated	$p < 0.01$	Intercept	-8.63** (2.92)	Soil N	0.15* (0.06)	SOC	0.02** (0.01)	Fine-root biomass	0.002** (0.001)	0.72
Pyrogenic carbon	Ambient	$p > 0.05$	Intercept	-0.77 (3.91)	Soil N	0.64 (0.33)					0.16
	Elevated	$p < 0.05$	Intercept	-0.96 (2.07)	Soil N	0.70** (0.20)	--	--	--	--	0.45
Lignin phenols	Ambient	$p < 0.01$	Intercept	71.11** (16.77)	SOC	-0.14** (0.04)	NPP	0.05** (0.01)	Water table depth	0.38*** (0.06)	0.80
	Elevated	$p < 0.01$	Intercept	56.87**** (7.42)	SOC	-0.06** (0.02)	Water table depth	-0.12** (0.03)	ANPP	0.01 (0.01)	0.72

Supplementary references

1. Hanson, P. J., Riggs, J. S., Robert Nettles, W., Krassovski, M. B. & Hook, L. A. SPRUCE whole ecosystems warming (WEW) environmental data beginning august 2015. *Oak Ridge National Laboratory, TES SFA, U.S. Department of Energy, Oak Ridge, Tennessee, U.S.A.* (2016) 10.3334/CDIAC/spruce.032.

2. Hanson, P. J. *et al.* Rapid net carbon loss from a whole-ecosystem warmed peatland. *AGU Advances* **1**, (2020).

3. Malhotra, A. *et al.* SPRUCE production and chemistry of newly-grown fine roots assessed using root ingrowth cores in spruce experimental plots beginning in 2014. *Oak Ridge National Laboratory, Oak Ridge, Tennessee, U.S.A.* (2020) 10.25581/spruce.077/1607860.
